# Tracking devices and physical performance analysis in team sports: a comprehensive framework for research—trends and future directions

**DOI:** 10.3389/fspor.2023.1284086

**Published:** 2023-11-23

**Authors:** António Ferraz, Pedro Duarte-Mendes, Hugo Sarmento, João Valente-Dos-Santos, Bruno Travassos

**Affiliations:** ^1^ CIFD, Sports Research, and Training Center, Jean Piaget University of Angola, Luanda, Angola; ^2^ Department of Sport Sciences, CIDESD, Research Center in Sports Sciences, Health Sciences, and Human Development, University of Beira Interior, Covilhã, Portugal; ^3^ KinesioLab – Research Unit in Human Movement, Institute of Piaget, Almada, Portugal; ^4^ Department of Sport and Well Being, Polytechnic Institute of Castelo Branco, Castelo Branco, Portugal; ^5^ Sport, Health and Exercise Research Unit—SHERU, Polytechnic Institute of Castelo Branco, Castelo Branco, Portugal; ^6^ Research Unit for Sport and Physical Activity, Faculty of Sport Sciences and Physical Education, University of Coimbra, Coimbra, Portugal; ^7^ CIDEFES, Centre for Research in Sport, Physical Education, Exercise and Health, Lusófona University, Lisbon, Portugal; ^8^ COD, Center of Sports Optimization, Sporting Clube de Portugal, Lisbon, Portugal; ^9^ Portugal Football Scholl, Portugal Football Federation, Lisbon, Portugal

**Keywords:** global positioning systems, local positioning systems, performance analysis, training and development, elite athletes

## Abstract

**Background:**

Tracking devices, such as global (GPS) and local (LPS) positioning systems, combined with physiological measurements, have become reliable tools to characterize movement patterns, assessing the external load (EL), internal load (IL), fatigue, and performance of athletes in team sports. This scoping review aims to provide a comprehensive understanding of the applicability of tracking systems in physical performance analysis within team sports and the wellbeing of athletes based on research strategies and combined variables.

**Methods:**

A systematic search was conducted in PubMed, Web of Knowledge, and Scopus databases according to PRISMA guidelines. The 79 studies that were reviewed met the following criteria: (1) contained relevant data regarding elite athletes′ performance; (2) athletes' EL and IL; (3) were written in the English language; (4) were related only to team sports.

**Results:**

The findings indicate that tracking technology has been engaged in several research areas, including performance analysis, training vs. match load management, injuries, and nutrition, through characterization and correlational studies. Metrics, primarily focused on kinematic and mechanical EL aspects, have been employed in combination with IL data to analyze the performance of athletes. However, the lack of an integrative model for the analysis and integration of EL and IL metrics within each team sport suggests an interesting direction for further research.

**Conclusion:**

There is a need for coherence between the methods and the research goals on performance analysis. The development of a framework that guides experimental studies is highly recommended, particularly on manipulating metrics analyzed between training and match sessions, injury prevention, and nutrition. This will lead to the development of the most applied sports science research to improve the preparation and decision-making of athletes based on reliable data.

**Systematic Review Registration:**

https://inplasy.com/?s=2022120039, identifier 2022120039.

## 1. Introduction

It is commonly recognized that team sports necessitate the development of various intense activities based on the space and time relationship between teammates and opponents, implying that movement production requires a perception–action relationship, in which short- and long-duration efforts are needed to achieve the most functional individual tactical actions (1). To achieve this, players must persistently adapt their field placement and enhance their preparedness to undertake actions with or without the ball by producing physical patterns such as accelerations, decelerations, changes in direction, short-term movements, and high impacts (1). Therefore, a precise and appropriate assessment of both internal load (IL) and external load (EL) considering the purpose of analysis is required to improve the understanding of the physical efforts and performance of players (2).

Regardless of some reservations about the quality, reliability, and validity of global positioning system (GPS) and local positioning system (LPS) measures (3), tracking athletes' time motion, combined with physiological measures such as heart rate, has become a common method for characterizing athletes' EL and IL (4, 5). An analysis of EL refers to quantifying players' movement demands regarding volume and intensity (6). According to the literature (7), EL can be divided into different categories such as (1) kinematics, which calculates overall movement during exercise; (2) mechanical, which describes the player's overall load during exercise; and (3) metabolic, which measures overall movement energy expenditure during exercise. In addition to the information provided by EL, athletes may experience varied physiological (IL) reactions (8). In this setting, IL is the individual athlete’s response induced by the EL stimulus, which is commonly determined by heart rate (HR) parameters (9). Thus, an investigation of the relationship between EL and IL units might be considered one of the most critical elements to monitor the dynamics of players' fatigue over the microcycle and their preparedness for a competition (10).

A lot of research has been developed in team sports over the last few years in order to characterize game demands and identify the most demanding passages of play, to understand the influence of different training manipulations during training sessions, or even to better understand injury mechanisms (11, 12). Also, some systematic reviews have been published in the last 2 years (4, 13, 14) with specific goals to understand the relationship between IL and EL or to characterize methods to collect and interpret EL (15). According to the literature, combining both information (IL and EL) allows one to perceive how workload promotes physiological impacts in players or comparing match/training sessions (5). As a result, the distinction of EL and IL units may reveal an athlete's level of exhaustion or enable the identification of the training plan. For example, it was already reported that the efficiency index (Eff_index_), which combines IL and EL, is a powerful tool for the evaluation of fitness status in team sports (14, 16). In addition, it has been emphasized that selecting appropriate metrics is crucial for accurately describing, planning, monitoring, and evaluating the training and competition aspects of each sport (16, 17). This information may aid in the management of training loads, thus lowering the risk of injury (4, 18) and improving athletic performance (4, 19). A systematization of prior research is essential to better understand the variables/forms of analysis that better explain the damage mechanism or performance improvement. However, to the best of our knowledge, no research has elucidated the relationship between various metrics, the intended purpose of the analysis, and its applicability for coaching and scientific purposes. An understanding of how the information from tracking technology devices has been used in previous research and what are the main issues that have been studied are required, to improve further research and practice. Thus, this scoping review aims to understand the applicability of tracking systems for physical performance analysis in team sports in the last decade by allowing an understanding of how research strategies and variables have been used and combined according to research goals. Based on this systematization, a comprehensive framework of physical performance analysis in team sports and athlete wellbeing is proposed.

## 2. Methods

Preferred Reporting Items for Systematic and Integrative Reviews and Meta-analysis Statements (PRISMA) were used to conduct a scoping review (20).

### 2.1. Search strategy: database and inclusion criteria

According to the PRISMA statement recommendations, a systematic search was undertaken on the electronic databases of PubMed, Web of Knowledge (all databases), and Scopus (20). A search of relevant articles published between January 2011 and July 2023 was conducted using the keywords [“global position system” OR GPS OR “local position system” OR LPS) AND (“team sports*,” Indoor team sports**”) AND (performance* OR “external load” OR “internal load”)]. The publications that were retrieved had to meet the following requirements: (1) should provide relevant statistics and research strategies about elite athletes' performance; (2) athletes' EL and IL; (3) published in English; and (4) should be only about team sports. Exclusion criteria applied if they (1) specifically pertained to the reliability, validity, or precision of global positioning system equipment; (2) pertained to systematic reviews, (3) were published before 2011.

To improve research accuracy, two reviewers (AF and BT) independently screened titles and abstracts to identify articles that would potentially combine the inclusion criteria after noting the features of each study, such as the authors' names, sample, procedure, and results or main outcomes. In the event of disagreement about the article's eligibility, a third reviewer (HS) was brought in to make a final judgment. Following a review of all papers, the study categories were divided into multiple sports depending on the primary research topic.

### 2.2. Quality of studies and data extraction

The quality of the studies was assessed in accordance with what is indicated by Faber et al. (21) and using the criteria for critical review forms recommended by Law et al. (22) (16 items) with the goal of finding studies in which low-quality scores could interfere with the results. Both researchers (AF and BT) separately assessed the quality of each eligible article. The interrater agreement was moderate (*k* = 0.43) (23). The possible criteria for each item were 1 (meets criteria), 0 (does not meet the criteria), or NA (not applicable). According to the methodology assessment, articles were evaluated with regard to their purpose (item 1), relevance of background literature (item 2), appropriateness of the study design (item 3), sample included (items 4 and 5), informed consent procedure (item 6), outcome measures (item 7), validity of measures (item 8), significance of results (item 9), details of intervention (item 10), analysis (item 11), clinical importance (item 12), description of drop-outs (item 13), conclusion (item 14), practical implications (item 15), and limitation (item 16). According to the guidelines of Faber et al. (21), a final score was determined, allowing the publications to be classified as (1) low methodological quality (50%), (2) good methodology quality (50%–75%), or (3) exceptional methodology quality (>75%). A data extraction sheet (based on the Cochrane Consumers and Communication Review Group's data extraction template) (24) was adjusted to meet the research inclusion criteria for this analysis and then evaluated on 10 randomly chosen studies (pilot test). The data were extracted by one author and validated by another. Disagreements were settled by discussions between these two authors (first and last). To organize the findings, the studies were divided into categories based on the major study issues.

## 3. Results

### 3.1. Search, selection, and inclusion of studies

**Figure 1:**
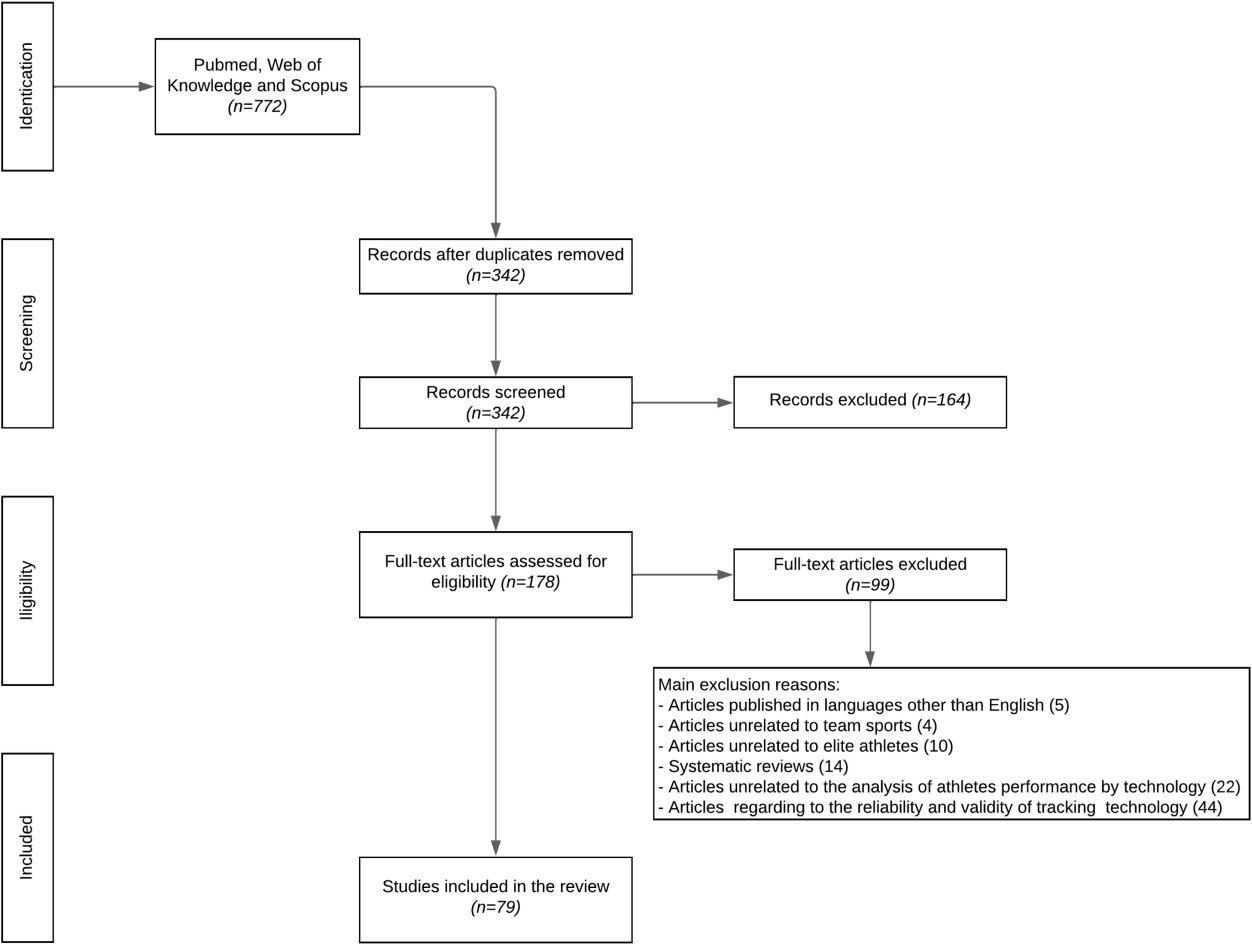
Flow chart of the article's selection process.

The first search generated 772 titles. These data were then exported to Mendeley (Elsevier, San Francisco, CA, USA), a reference manager software. Duplicates (*n* = 430) were either automatically or manually removed. The remaining 342 titles were then evaluated for relevance using information from their title and abstract, with 164 being eliminated. The remaining 178 papers were examined further, and 99 were excluded based on the following criteria: (1) articles published in languages other than English (*n *= 5); (2) articles unrelated to team sports (*n *= 4); (3) articles unrelated to elite athletes (*n *= 10); (4) systematic reviews (*n *= 14); (5) articles unrelated to the analysis of athletes' performance by technology (*n* = 22); and (6) articles concerning the reliability and validation of tracking technology (*n* = 44). The review comprised 79 studies in total (Figure 1).

To understand the scope of the development of research methodologies in physical performance evaluation in team sports during the last decade, the publication target years were restricted from January 2011 to July 2023. The articles used for in-depth reading revealed that there had been an increase in GPS and LPS research. In this review, 84.8% of the studies were published in the last 5 years (from 2017 to 2023) and 15.2% between 2011 and 2016. On average, 6.3 studies were published per year.

### 3.2. Quality of the studies

With regard to the quality of the studies, following the proposal of Faber et al. (21), the average score of the 79 selected studies was 89.2%. Fourteen studies achieved 100%; 52 achieved an average rating of ≥75%; 12 achieved a score between 50% and 75%.

### 3.3. General description of the studies

Studies were categorized by the sport in which the studies were carried out: (1) football (FTB) (41.8%), (2) futsal (FTS) (15.2%), (3) rugby (RG) (11.4%), (4) Australian football (AUF) (7.6%), (5) field hockey (FH) (6.3%), (6) Gaelic football (GF) (6.3%), (7) basketball (BK) (3.8%), (8) American football (AMF) (2.5%), (9) ice hockey (IH) (2.5%), and (10) rink hockey (RH) (2.5%).

### 3.4. Variables and goals of the studies

**Figure 2:**
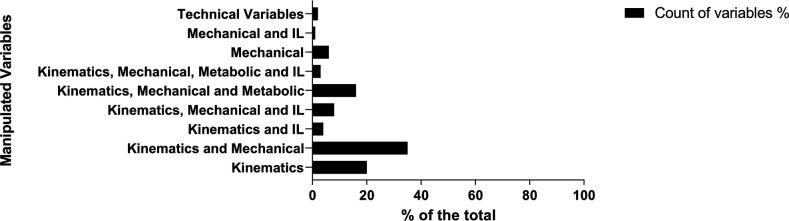
Distribution of grouping variables monitored by the study.

The studies were classified based on the criteria used in the study. Most of the team sports research employed tracking technology: (1) combined kinematic and mechanical variables (35.4%); (2) only considered kinematics (21.0%); and (3) included kinematic, mechanical, and metabolic variables (16.5%). On the other hand, the analysis of IL or the relationship between mechanical variables and IL variables was less explored (Figure 2).

**Figure 3:**
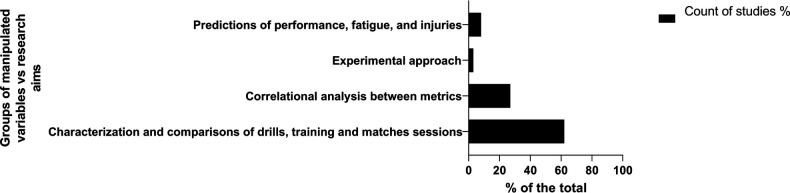
Distribution of studies according to study results.

Studies were also grouped according to the type of analysis of the studies. The results revealed that the studies were distributed by using the following analyses: (1) characterization and comparison of drills, training, and matches sessions (62.0%), (2) correlation of variables to understand their relationship in training and competition (26.6%), (3) predicting performance, fatigue, and injuries (8.7%), and finally, (4) experimental studies to determine greater training and team management approach (2.7%) (Figure 3).

**Figure 4:**
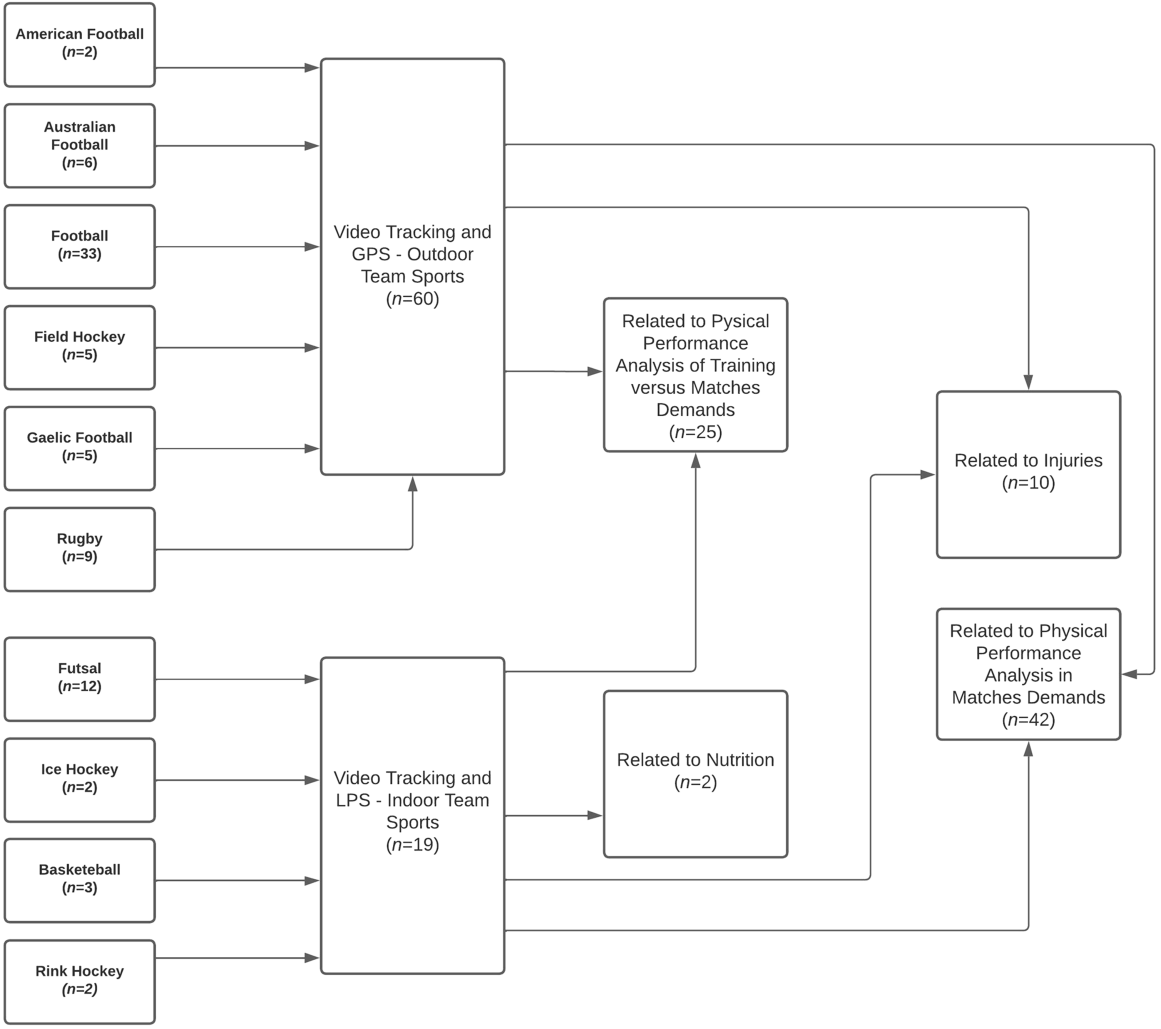
Flow chart of the related sports and leading GPS and LPS research topics in past years.

With regard to the main research topics from the studies in analyses, the following was observed: (1) performance analysis in match demands, (2) performance analysis of training vs. match demands, (3) injuries, and (4) nutrition (Figure 4).

The studies reviewed provide information on the use of tracking systems in various domains of sports sciences, such as the importance of technology in better understanding the physical requirements for coadaptations between match demand information in which players establish interactions with teammates and opponents in order to manipulate training sessions and, as a result, to improve athletes' awareness and physical performance (25, 26). In addition, studies were used to determine whether specialized nutritional intake could improve players' responses to the demands of an indoor team sport (27, 28). Also, in recent years, multiple sports have used various methods to track injuries by understanding the external information that better characterizes sporting activities (7, 29, 30).

With regard to the understanding of training and match demands in the performance and analysis of team sports, several studies have been carried out in order to compare and understand positional (31), tactical, and technical differences (32), intragame information (33, 34), comparisons of the levels of competitions (35), predictors of intensity, fatigue, and match performance (36–39), and more recently, to understand the games’ most high-intensity activities (HIAs) and periods (MIPs) (36, 40) (Figure 4).

### 3.5. Applicability of the tracking system related to performance analysis in match demands

With regard to the performance analysis of match demands, there are several studies involving the characterization and understanding of EL metrics in various team sports (32, 41–46) as well as its impact on athletes’ IL (36, 47–51). Some studies describe a moderate to high correlation observed between EL and IL (38, 48). In addition, there are studies using tracking systems in which the main goal is to find correlations between metrics; (1) kinematic vs. mechanical (36, 43, 52); (2) kinematic vs. mechanical and metabolic (37, 41).

A study with 118 elite male ruby league players shows significant increases in the offensive and defensive tackles between senior and junior players and between forwards and backs (53). In the same study, independent of the playing level, running demands (HSR, ACC, DEC) were significantly greater in backs than in forwards (53). There is a high prevalence of studies in which EL metrics are classified based on player level and player position; positioning is often related to an event or to the magnitude of EL metrics (33, 46, 53–56). However, positioning associations are not always found in team sports. In a study of nine elite RH players, there were no significant differences throughout all observed metrics during a competition between players' positions (57). Furthermore, variable load distribution (total PL) based on the axis of movement was not verified based on playing position (58). However, total PL appears suitable for monitoring locomotor demands or accelerometer-derived load in FTB (58).

Finally, and more recently, studies seem to aim for the prediction and proposal of models of fatigue (59) and match performance according to match demand information (44, 60–63) (Table 1).

**Table 1:** Articles predominantly related to match physical performance analysis.

Study and sport in which the study was carried out	Sample	Main outcomes measured	Results	Quality score (%)
(64)—Rugby	8 elite female players (1) Backs (*n *= 4) age, 27.0 ± 2.6; (2) Forwards (*n *= 4), age 26.6 ± 1.9	Heart rate (HR), time, speed (SP), distance (DT), location, and number and intensity of impacts (Himpts) and accelerations (ACC) expressed as g forces). GPS units were used.	The players covered 5,820 ± 512 meters and showed positional cinematic and mechanical differences. They exerted themselves at 91%–100% heart rate for 46.9% ± 28.9% of the match.	96.7
(65)—Gaelic Football	56 elite male players (age, 15 ± 0.66 years; height, 176.8 ± 6.5 cm; weight, 69.08 ± 6.72 kg and VO2max 50.77 mL-kg−1min−1)	Predicted VO2max was assessed using the Yo-Yo Intermittent Recovery Test. Players HR, Speed zones, total DT, and the number of sprints were recorded by GPS units.	Players’ intensity levels peaked at 85% of match maximum HR. The distance covered in the second half was less than that in the first, and midfielders covered the most ground at 6,740 ± 384 meters.	90.0
(43)—Rugby	33 elite male players (age: 25.2 ± 3.5 years; weight: 101.2 ± 13.2 kg; height: 79.8 ± 33.0 cm	ACC, DEC, and body impacts during match play were recorded using GPS units.	Forwards had intense impacts and greater physical demands, while backs had frequent moderate to heavy accelerations and decelerations.	93.3
(66)—Gaelic Football	50 elite male players: age (24 ± 4 years), height (180 ± 7 cm), weight (81 ± 7 kg), and years on squad (5 ± 3 years)	The match's performance was tracked using GPS, measuring distance covered, speed, ACCs, and peak velocity.	Players completed an average of 2.6 ± 0.5 ACC per minute. Midfielders covered more total and high-speed running distance. Running performance reductions varied by player position.	90.0
(46)—Football	46 elite players (age 20 ± 3 years, height of 179 ± 5 cm, body mass of 79.5 ± 6.3 kg)	GPS units were used to assess total distance (TD), high-speed running (HSR), high metabolic load distance (HMLD), ACC, and DEC	Different formations affect player performance. The 3-5-2 has high TD and HMLD, while the 4-2-3-1 has high ACC and DEC.	80
(67)—Football	A total of 2,951 rows from training and official matches of 42 elite players	GPS devices were used to collect (1) Locomotor Variables, (2) Metabolic Measures, and (3) Mechanical Variables.	Certain variables can represent as representatives of a collection of highly related variables, lowering the number of variables required in coaches’ periodic physical analyses from 17 to 4.	66.7
(52)—Rugby	63 players of the French or Irish national u20 teams. (age: 19.8 ± 0.5 years, body mass: 99.1 ± 9.1 kg, stature: 185.4 ± 7.0 cm)	GPS devices tracked running abilities: TDm.min 1, HSR m.min 1, HMLD m.min 1, Sprints n.min 1, ACC n.min 1.	Higher metabolic loads in back and forward players. Players with the most match-play exposure exhibited moderate-to-large decreases in total and HMLD in backs.	90.0
(25)—Australian Football	39 elite athletes (age: 23 ± 4 years, height: 187 ± 8 cm, mass: 86 ± 9 kg).	For all indoor matches, athlete physical output was collected via the local positioning system (LPS). During outdoor matches, all participants wore a GPS device.	Two methods were developed to identify the relationship between physical, skilled, and temporal outputs, on and individual and team level.	93.7
(59)—Football	22 elite players (age = 21.96 ± 4.53 years; height = 180.68 ± 5.23 cm; weight = 72.36 ± 4.19)	RPE and Training and Match Workload—21 kinematic, 37 metabolic, and 30 mechanical metrics) were computed from the GPS raw data.	Results suggest that it is possible to predict RPE from GPS training and match data.	80.0
(68) –Gaelic Football	85 Gaelic football players (U-18) (17.57 ± 0.53 years)	Anthropometry (% body fat), Vo2Max and Match running activity categories with GPS devices.	Players cover an average of 5,774 ± 737 m in a 60 min match and achieve % HRmax (81.6 ± 4.3%) and %VO2max (70.1 ± 7.75%). There are positional differences.	87.5
(32)—Gaelic Football	432 individual full match datasets collected across 52 matches of top teams.	Technical variables; Total distance (m) and high-speed distance (≥17 km h−1 m) with GPS units and minutes spent on the pitch.	Ball play duration, opposing team's short kick-outs, and possession time impact overall and high-speed distance run.	86.7
(69)—Football	6 professional players (23.0 ± 1.8 years)	Velocity and subsequently GPS acceleration data that were used to quantify player movement.	Shorter durations showed inconsistencies in high-speed runs, sprints, and ACC. Longer minimum durations led to a decrease in efforts.	86.7
(70)—Field Hockey	10 male (age; 38.7 ± 5.4 years, stature; 1.77 ± 0.07 m) and 11 female (age, 33.5 ± 6.5 years; stature; 1.66 ± 0.04 m)	Effort frequency, distances, and time were measured by using GPS units	Differences between males and females in total distance covered, time spent engaged in high-intensity running (HIR), frequency of HIR, and distance covered during each HIR effort.	86.7
(53)—Rugby	118 elite male players (57 seniors aged 28.7 ± 4.4 years; 61 juniors aged 17.2 ± 0.5 years)	Positional game demands using a GPS unit and video analysis. The anthropometric, locomotor, and contact metrics were recorded.	Senior and junior players, including forwards and backs, have improved in tackles. Backs have higher kinematic and mechanical metrics than forwards	93.3
(54)—Rugby	38 elite players (23 ± 3 years;1.87 ± 0.06 m; 99 ± 10 kg)	Heart rate training impulse (HR-TRIMP), sRPE-TL. GPS metrics were monitored: Mechanical Work; Impulse, Metabolic Work; HP Distance; ACC/DEC; HS Distance; Distance.	EL correlates strongly with IL indicators. Total effective load (TEL) is best determined by combining HR-TRIMP and EL, considering body weight and ACC/DEC.	100
(55)—Football	5 matches and 60 players from Professional First Italian League.	Evaluate speed, acceleration, deceleration, and metabolic power in soccer players through video tracking (K-Sport Universal, Italy).	Most frequent events occur within 1–2 m of ACC and DEC threshold. External midfielders perform the highest-intensity actions.	53.3
(35)—Rugby	188 professional players from teams from Ireland, Italy, Scotland, and Wales.	Positioning metrics using GPS-IMU: TD (m), meterage (m.min−1), HSR; maximum velocity (m.s-1); number of efforts distance and repeated high-intensity locomotive efforts (RHILE) and HSR efforts	RHILE was higher in international games than in club games; out-side backs (OB) showed greater distance and meterage in international games. Significant differences were observed across all six positional groupings (*P* < 0.05).	60.0
(33)—Basketball	94 elite under 18-year-old basketball players (age, 17.6 ± 0.8 years; height, 1.91 ± 0.08 m; Body mass, 82.5 ± 8.8 kg; BMI, 22.7 ± 1.8 kg/m^2^)	Time spent on activities during a soccer match for positional differences. EL using GPS technology: distance covered, player load (PL), ACC/DEC, peak speed, and peak acceleration.	Top teams had lower RD, with guards having higher RD than forwards and centers. First quarter showed higher RD, %HIR, and PL. Third match had higher demands in RD, HIR, and PL than the first two matches.	100
(71)—American Football	43 collegiate players (age, 19.9 ± 1.5 years)	DT (m); maximum velocity (MV) total inertial movement analysis (Total IMA) = ACC, DEC. Positional differences [Wild receivers (WRs); Defensive backs (DBs); Offensive line (OL); Defensive line (DL).	DBs traveled farthest, but DLs exceeded them. MV found that DLs scored higher than OLs. WRs had the strongest acceleration. All positions differed in DEC intensity.	86.7
(72)—Ice Hockey	20 elite male players. Seven defenders (age; 19.3 ± 0.5) and 13 forward players (age 19.3 ± 0.7)	Skating speed thresholds were monitored by period (1°; 2°, 3°) and by game situation (5v5; 4v5; 5v4):	During gameplay, forwards have more high-intensity skating than defenders, while both positions experience a decrease in skating intensity during the third period.	86.7
(38)—Football	18 male players (seven defenders, five midfielders, and six attackers) from the Norwegian Premier League (age, 26 years; height 183 cm, body mass 80 kg)	PL, layer load; HIE, high-intensity events; HSRD, high-speed running distance; sRPE-TL, session rating of perceived exertion training load; VHSRD, the very high-speed running distance by the GPS.	Total distance, PlayerLoad™, PlayerLoad2D™, and HIE >1.5 had most likely substantial within-player effects on sRPE-TL. sRPE-TL showed large to very large between-session variability with EL.	90.0
(73)—Football	23 female elite players (age: 27.65 ± 4.66 years; height: 165.35 ± 5.82 cm; weight: 60.91 ± 5.34 kg).	EL [walking distance (WD, m); jogging distance (JD, m); running distance (RD, m); sprinting distance (SD, m); different zones of ACC/DEC, were assessed by players’ positioning across different matches.	Decrease in external locomotor demands across played matches.	93.3
(37)—Futsal	28 elite male players (age: 24.1 ± 3.4 years) from eight futsal teams from the Final Eight of the Portuguese Cup 2018	The GPS measured various metrics, including distance covered, sprints, maximum speed, impacts, jumps, stress load, and metabolic power.	Player performance was assessed based on kinematic and metabolic metrics. The strongest correlation was found between cluster levels, DEC, and an increase in DT per minute in the second half.	96.7
(61)—Futsal	13 elite players (age: 28.8 ± 2.4 years, weight: 73.7 ± 6.2 kg, height: 175.9 ± 5.9 cm)	With GPS units, kinematics (absolute high-speed running, relative speed running, and total distance) and mechanical (PL), high-intensity ACC and DEC) were assessed.	MD-2 is similar to the match. High- and very high-demanding scenarios (n) in the training session prior to the match dropped in comparison with the rest of the microcycle and the match.	93.3
(74)—Futsal	14 elite players (age,30.21 ± 3.98 years; height, 1.77 ± 0.07 m; weight, 74.85 ± 6.40 kg) from a professional club of Spanish Futsal first division League.	The LPS monitored various metrics: distance, ACC, and velocity. These included Relative Distance, Explosive Distance (ED), HSR, ACC, DEC, ACCMAX, DECMAX, ACCMEAN, DECMEAN, Velocity MEAN, and Sprints.	Physical requirements provided similar outcomes in the first and second halves. Wingers outperformed pivots in terms of HSR distance. All player positions had a high amount of ACC and DEC each minute.	93.3
(36)—Futsal	16 elite male futsal players (age, 25.74 ± 4.71 years; body mass, 74.2 ± 9.8 kg; body fat 11.1 ± 5.8%)	Maximum Heart Rate (MHR) by Yo-Yo Intermittent Recovery Test. In Competition: Internal measures (Heart rate); EL: Low Medium and High ACC per/min; PL (a.u). Technical Variables	MHR via the Yo-Yo IR1 test (194.6 ± 11.1 beats min−1). Mean HR value during “court time” of 164.7 ± 22.3 beats min−1. 77.3% of ball receptions were completed with the sole of the foot. 80.1 ± 16.7% of individual possessions used the dominant foot to receive and 84.1 ± 10.7% to pass the ball.	73.3
(62)—Futsal	87 male U17 athletes (three to five sessions of weekly training) and 85 elite adult male athletes (8 to 12 sessions of weekly training).	Video tracking to record % very HIR, total distance covered (TDC)/min, successful passes, pass efficiency, and substitutions in each half.	The number of substitutions contributed to higher TDC, %VHIR. Decrease in %VHIR promoted lower pass efficiency. Substitution improved running performance.	93.3
(75)—Futsal	79 top-league adult players (age: 28.4 ± 4.6 years) and 59 top-level youth players (age: 17.1 ± 0.7 years) i	Percentage of distance covered (%). Speed categories: walking (0–6 km/h), low-intensity running (LIR; 6.1–12 km/h), medium-intensity running (MIR; 12.1–15.4 km/h), HIR; 15.5–18.3 km/h, and sprint (>18.4 km/h). High-intensity exercise (HIE) = sum of MIR, HIR, and sprint.	Youth players have longer playing time and lower HIE than adult players in sports. Pivot players cover less ground than wingers in adults, and defenders have lower levels of HIE without ball possession than wingers.	86.7
(76)—Futsal	43 elite male futsal players from six elite futsal teams	Warm-up routines: Closed skills, Open skills, and Futsal-specific skills. EL was monitored by using the LPS: Total distance covered (m); Distance covered (m/min); running (m/min); Sprinting (m/min); ACC (n/min); DEC (n/min)	Including futsal-specific warm-up tasks prepareplayers for the game. ACC and DEC increase during warm-up, leading to higher intensity.	73.3
(63)—Football	19 football players (age, 26.78 ± 3.77 years old; body mass index, 23.1 ± 0.19) from the La Liga.	GPS units to track the worst-case scenario (WCS): TD covered; HSR distance (HSRD) and sprint distance (SPD). The WCS were generated using fixed length and rolling average approaches based on playing position.	Fixed length methods of varying durations greatly underestimated the WCS of TD, HSRD, and SPD across playing positions. In professional football match play, the rolling average approach is suggested for obtaining a reliable WCS analysis.	86.7
(77)—Field Hockey	24 international hockey players from an international hockey team (age = 26 ± 4, max aerobic speed = 4.85 ± 0.23 m.s−1).	EL by the GPS: Relative total distance (RTD); HSR; Sprints distance (SD); ACC; DEC; Low-speed running (LSR), Dynamics stress load (DSL); ED, HML efforts; HML distance (HMLD); Total Load (TL)	Significant effects were found for possession status on several physical output metrics. Not possession, except for forwards, is the category with more demand (RTD, ED, HSR).	93.3
(78)—Football	26 male professional players (age: 28 ± 4 years; height: 182 ± 6 cm; body mass: 78.8 ± 6.2 kg).	Optical tracking to determine de WCS for TD, high-speed running (>5.5 m.s−1) and sprinting (>7.0 m-s−1).	The WCS, defined as the maximal physical load in a given time-window, produces unstable metrics lacking in context, with high variability.	86.7
(79)—Futsal	14 professional players (age: 28.8 ± 2.4 years, weight: 73.7 ± 6.2 kg, height: 175.9 ± 5.9 cm)	LPS to measure EL by playing position: Relative distance (m.min−1), HSR distance (m); HSR efforts > 18 km.h−1 (n); ACC (>2m.s−2; n.min−1), DEC (>2m.s−2; n.min−1); ACC distance (>2 m.s−2; m.min−1); DEC distance (>2m.s−2; m.min−1).	EL load metrics vary among positions and players. Contextual factors can affect the perceived difficulty of demanding scenarios.	100
(80)—Football	25 male professional football players from the German Bundesliga.	GPS tracking to analyze the physical match performance; total distance, high intensity distance (17–23.99 km/h), sprinting distance (≥24 km/h), accelerations (≥1.5 s) for each player and each position.	The change in physical match performance can be explained by 44%–58% through the normative positional data. Individual differences impact the way players perform when acting in different positions.	86.7
(34)—Rugby	11 Elite national team players (age: 24.3 ± 3.3; stature 166.1 ± 7.2 cm; body mass: 66.1 ± 7.4 kg)	GPS units to track EL measures: TD/min; Standing/walking (0–6.0 km.h −1); jogging (6.1–12.0 km.h −1) Cruising (12.1–14.0 km.h −1); striding (14–1-18.0 km.h −1); HIR (18.1–20.0 km.h −1); sprinting (>20.1 km.h −1); ACC(n) (>1.8 m.s−2) and DEC(n)(< −1.8 m.s−2). IL was by s_RPE and wellbeing.	No significant differences between congested matches were observed in almost load measures. Congested match schedules negatively impact RPE, muscle soreness and overall wellness.	100
(81)—Futsal	126 professional players, including goalkeepers 6.	Video tracking system (30 Hz) to measure distance covered (DC) and percentage of DC in different speed ranges to identify differences per team and per subphase of the game (traditional vs. outfield goalkeeper situation.	With the outfield goalkeeper situation, the team spent a higher percentage of the distance covered in the standing and walking speed range compared with the traditional goalkeeper positioning.	90.1
(40)—Futsal	17 male professional players (age: 28.8 ± 2.4 years, weight: 73.7 ± 6.2 kg)	WIMU LPS to assess high-intensity activities (HIAs: sum of acceleration, decelerations, and high-speed running actions).	Players with more playing time and with a specific work-rest ratio (1.1 ± 0.6 a.u.) show a greater ability to repeat HIAs per rotation.	100
(82)—Football	20 elite players (age, 29.4 ± 4.4 years; height, 1.8 ± 0.1 m; and body mass, 74.8 ± 2.3 kg)	Chronic workload ratio (ACWR) via session-rated perceived exertion (s-RPE). GPS units tracked DC, HSR, and SPD by starters and non-starters during the season.	ACWR was the highest at the beginning and end of the midseason, with higher values at the start of the early season, specifically through SPD. Correlations were found between EL and IL metrics across all three in-season periods.	96.7
(83)—Football	14 professional male players (age: 23.86 ± 3.58 years; weight: 73.74 ± 5.92 kg, height: 1.79 ± 0.05 m)	Maximum intensity periods (MIPs) tracked by using GPS units: distance covered at HSR, and Sprinting, ACC density (AccDens), mean metabolic power (MetPow), meters per minute (Mmin) and HMLD >25.5 W/kg.	Differences in HSR, Sprint, AccDens, MetPow, and HMLD thresholds between players. Positional variations were observed in MetPow, Mnin, and between halves in AccDens, MetPow, and Mmin.	93.3
(56, 84)—Football	20 elite players (age: 29.40 ± 4.35 years; body mass: 75.00 ± 3.87 kg; height: 1.79 ± 0.05 m; body mass index: 23.38 ± 1.79).	ACWR and exponentially weighted moving average (EWMA) were calculated using session-rated perceived exertion (s-RPE), total distance (TD), HSR distance (HSRD), and SPD during three seasons. Kinematic metrics were evaluated using GPS devices.	Except for EWMAsprint, workload measures observed in the midseason were higher than those in the early season. Wingers and strikers tended to have a greater workload than defenders and midfielders.	96.7
(58)—Football	19 male elite players (26.8 ± 3.8 years, 1.79 ± 0.08 m, 73.6 ± 6.4 kg)	PL (Tri-axial) with the GPS (WIMU tracking system) by player's position.	Total PL may be suitable for tracking locomotor demands or accelerometer-derived loads.	96.7
(85)—Football	17 elite young players (15.2 ± 0.3 years, 171.4 ± 6.5 cm, 62.5 ± 7.5 kg)	During specific SSGs, EL such as TDC moderate speed running (MSR), HSR, Sprinting running (m), ACC/DEC (n), and PL, was assessed by using the GPS. MHR and RPE were also assessed	In SSGs, shorter defensive periods enhanced HSR, while longer defensive periods raised RPE.	100

### 3.6. Applicability of the tracking system related to the performance analysis of training vs. match demands

Based on an analysis of the selected studies, at the same level, studies suggest that it is possible to predict IL such as RPE from GPS training and match data (59), showing that the variation in workload efficiency throughout matches may be explained by the training load variables from the prior days and training cycles (60). In addition, an understanding of load dynamics across training and competition is presented as an important aspect related to performance enhancement in team sports (8, 47, 60, 61), showing, for example, how adjusting specific drills such as large,- medium- and small-sided games may help replicate game demands (86, 87). Correlations between EL and IL in different season periods were applied in order to understand the fluctuation of some metrics such as players’ strength (88), players’ recovery (89), or ACWR, between players’ positions, starters, and non-starters (82, 84, 90, 91). Also, Pizarro et al. (26) demonstrated how different occupational areas may promote different physical variables. The authors state how the manipulation of “floaters” in SSGs according to specific purposes in FTS promotes higher distances and speed variables, accelerations, and decelerations. The same was described by De Jong et al. (81), in which manipulation of the game momentum (with the outfield goalkeeper) promotes changes in team total distances covered (81). Silva et al. (92) demonstrated that teams have the ability to coadapt to performance constraints by manipulating SSGs. In addition, add-ons to SSGs, such as the number of players by drill, player positioning, or touch constraints, are being referenced as potential ways to increase or decrease EL (81, 93) (Table 2).

**Table 2:** Articles predominantly related to training versus match physical performance analysis.

Study and sport in which the study was carried out	Sample	Main outcomes measured	Results	Quality score (%)
(56)—Australian Football	2,700 training or competition GPS recordings from 44 athletes.	Time spent at each speed was analyzed using the GPS, by: (1) Game vs. Training Comparison, (2) Player Comparison, (3) Intraseason and (4) Position Comparison.	A strong correlation between the intensities of the training sessions and the physical demand of first grade games and small differences in the demand of different field positions was also observed.	53.3
(45)—Field Hockey	44 elite male players (age 27.0 ± 2.7 years, body mass 78.8 ± 6.8 kg)	The Fisher *Z*-test was used to analyze the relationships between distance and PL in absolute (m, AU) and relative (m/min, AU/min) terms between matches and training, as well as between positions within matches.	In competition, the absolute distance–PL relationship was very large overall, with no variances between positions. The relative distance–PL relationship was moderate overall, but weaker in forwards than in defensive midfielders’ and defenders. In training, the absolute/relative distance–PL relationship was very large.	100
(48) –Australian Football	44 professional elite athletes (mean ± SD: age, 24.1 ± 3.8 years; height, 187.7 ± 7,2 cm; body mass, 87.3 ± 8.2 kg)	IL data: RPE-based method and s-RPE. GPS units to obtain EL: total distance (m), HIR (>14.4 km/h (m) (HIR), PL and average movement speed (m/min).	During the preseason, the RPE load was greater. The RPE load was significantly reduced in the final preseason block. From preseason to in-season, TD, HIR, and PL showed decreases.	93.3
(94)—Rugby	Performance analysts/strength and conditioning coaches from (*n *= 3) Super Lead clubs in the North of England.	Michel Foucault's disciplinary analysis was used to explore how teams use wearable GPS technology and how it affected the physical, psychological, and emotional health of rugby football players.	GPS data can be used as a disciplinary tool to normalize and pressure athletes into complying with possibly harmful physical and physiological demands. When mismanaged, it enhances athletes' uncertainty, fear, and failure of the key elite demands.	71.4
(41) —Football	A total of 2,951 rows from training sessions (2,478) and official match (473) of 42 elite players throughout the 2015–2016 season.	GPS devices were used to collect (1) Locomotor Variables, (2) Metabolic Measures, and (3) Mechanical Variables.	The use of summarized physical variation data provided a relation between higher magnitudes of variation in 3-week time frames during training, and higher physical values in the following matches.	73.3
(51)—Football	22 elite female players (age = 28.3; Stature = 168.6; Body Mass = 64.3; % Body Fat = 18.8)	IL [heart rate metrics, ratings of perceived exertion (RPE), wellness ratings] and EL ([HSR, VHSR, and maximal sprint speed were monitored (GPS)].	Correlations between HSR and VHSR vs. RPE were large; VHSR for the MSS technique was moderate; HSR was highly associated with heart rate indices and was large for MSS.	86.7
(44)—Football	6 high-level female players (weight 59.6 ± 6.8 kg, height 171.5 ± 4.2 cm)	High-intensity run and sprints were monitored between players’ positions using GPS tags on training and match sessions.	In a normal microcycle, full backs covered only 26% of the SPD they covered in the next match. Practitioners must carefully consider proximity size and physical work pattern in microcycles to better resemble match performance.	73.3
(95)—Football	150 players, aged between 18 and 23 years across four seasons (2014–2018)	GPS tracking to profile high-speed running (HSR) activity and distance covered (DIST) between training and match sessions and playing position.	Positional differences during games. Thus, the physical activity profile does not show positional differences during training sessions. Central Forwards face the highest demands for HSRs. Central Midfielders tend to cover more distances.	86.7
(50)—Football	22 elite players (age = 21.96 ± 4.53 years; height = 180.68 ± 5.23 cm; weight = 72.36 ± 4.19 kg)	EL by the GPS; kinematic and mechanical metrics. IL; RPE and s_RPE.	RPE and S-RPE are more affected by training volume than intensity. RPE is affected by the previous training week workload. s-RPE reflects the workload performed in the current training session.	86.7
(96)—Rugby	22 elite rugby players (age; 25.7 ± 4.1 years, body mass;104.6 ± 12.6 kg).	Resting metabolic Rate (RMR); IL: RPE/s-RPE. EL: total distance covered, high-speed efforts/(n) and very high speed efforts/(n) were recorded on match (GPS).	Elite rugby matches can increase resting metabolic rate for up to 3 days after the game due to collisions during play.	87.5
(97)—Rink Hockey	8 professional male players (age: 29.6 ± 5 years; weight: 78.1 ± 4.6 kg; height: 178.8 ± 3.1 cm)	Using the LPS, the following EL metrics were monitored: DT; High-speed skating, HSS; ACC; DEC; and PL by players’ positions: Exterior (EX); Interior (IN).	There were no significant differences between positions in all monitored metrics.	93.3
(98)—Football	13 professional players (aged, 26 ± 4.6 years; body mass, 77.4 ± 6.96 kg; height, 181 ± 50 cm; body fat, 8.7 ± 1.51%)	Training and matches positioning differences: IL: Intensity zones of Heart rate (%HR max); EL: DT with specific running speeds; number of sprints; ACC/DEC (n).	Higher HR in matches. Fullbacks, central midfielders, and defenders showed higher HR. Low running intensities are higher in training that in games. ACC and DEC are higher in training sessions than official matches.	93.3
(60)—Football	14 professional soccer players age, 22.6 ± 4.3 years; height, 181.5 ± 5.7 cm; weight, 75.7 ± 5.8 kg) from the Austrian Second League	The GPS was used to track total distance (m); High, Very high, and SPD (m); N of medium ACC/DEC (n); N of high ACC/DEC (n). The modified training impulse (TRIMPMOD) was also obtained.	Previous day's training load affects workload efficiency in subsequent matches. Long sprints on D-3 and D-4, and total distance on D-1 impact workload efficiency. Sprint training on D-4 and D-3 positively influences workload efficiency in matches.	86.7
(39)—Australian Football	45 professional players (age: 24.95 ± 4.45 years; height: 1.87 ± 0.05 m; body mass: 84.64 ± 9.01 kg)	Training and match EL (TD; HSR and VHSR) measured with GPS units. IL assessed by s-RPE. Perceptual wellness (soreness, sleep, fatigue, stress, and motivation) questionnaire.	Principal Component Analysis (PCA) identified eight uncorrelated components from 37 monitoring variables. Seven-day TD; inertial movement analysis (IMA) event count and s-RPE load presented positive relationships with performance.	90.0
(47)—Rink Hockey	9 elite professional players (age: 29.8 ± 5.77 years, weight: 79.5 ± 5.50 kg, height 180.4 ± 4.03 cm)	GPS tracking for EL; DT (m); HSS (m); PL (a.u.) ACC (n); DEC (n) and IL (RPE and s-RPE).	MD-4 and MD-1 sessions with lower EL and IL. MD-3, MD-2 and MD presented higher external and IL with values near the fatigue zone. Loa dynamics tended to show an inverted “U-shape.”	93.3
(8)—Ice Hockey	17 elite male players (six defenseman and 11 forwards); age = 26 ± 5 years old, height = 181 ± 6 cm, body mass = 81.6 ± 6.9 kg, body fat = 13.6% ± 2.2%.	GPS tracking for EL: ACC variables (n/per minute) included the number of total ACC (ACCtot), ACC above 2 m·s−1 (ACC2), total DEC (DECtot) and DEC below −2 m·s−1 (Dec2). IL: HRmean, HRpeak, Edwards TL and TRIMPMOD, RPE and s-RPE.	ACC2 and DEC2, time spent >85% HRmean, Edwards TL and TRIMPMOD, RPE and s-RPE were greater during competition than during training; however, they decreased toward game day. ACCtot and DECtot per minute were lower than competition.	93.3
(63)—Football	30 male professional players (age: 25.97 ± 3.73 years old; height: 1.80 ± 0.07 m; weight: 74.60 ± 6.61 kg)	GPS tracking to EL metrics (mechanical, kinematic, and metabolic) were collected on both training and match days (MD)	Metabolic power, total steps, and various distances covered were key factors in explaining the PCA. −1MD and +1MD had the lowest load variability compared with −5MD and −4MD.	100
(26)—Futsal	30 male futsal players from the U-19 (age, 17.7 ± 0.71) from Spanish clubs.	The LPS was used to analyze EL (kinematics and mechanical variables) and IL (heart rate) by manipulation of floaters in four different contexts of 3vs3 Small-sided Games (SSGs).	Differences in the physical variables were observed. “Goal line Floaters” is related to higher distance and speed variables, being the most demanding SSG. Lower HR values were obtained, and “Floaters off” is linked to the ACC and DEC variables.	93.3
(93)—Football	20 professional soccer players (age: 28.1 ± 4.6 years; height 176.7 ± 4.9 cm; %BF 10.3 ± 3.8%)	GPS units were used to measure TD, HIR, HSR, and mechanical work (MW) in small-sided games (3v3, 4v4, and 6v6 formats).	TD min-1 showed consistency in EL metrics across SSGs, with only minor variations between intervals observed in HIR-1, HSR min-1, and MW min. Limiting touches or adding goalkeepers did not cause significant changes in session-to-session differences.	90.1
(49)—Football	29 elite male players (age: 18.3 ± 0.5 years, height: 175 ± 6 cm, weight: 65.5 ± 6.3 kg)	Small-sided games (8v8, 5v5, 3v3) with GPS units to measure maximum speed, distance covered, HSR, ACC, and DEC. HR bands and blood lactate to measure IL.	Players covered more distance and high-speed running in 8v8 and 5v5 compared to the 3v3 format. HSR distance was higher in 8v8.	93.3
(88)—Football	18 Brazilian elite football players (24.3 ± 4.8 years; 180.0 ± 5.7 cm; 74.7 ± 8.3 kg).	Training loads with GPS units, measuring EL and IL variables, as well as assessing vertical jump performance during a 4-week preseason.	High internal and external training loads during the preseason affected recovery and reduced jump performance, particularly in the group with higher VJ ability.	93.3
(89)—Football	25 U16-U17 youth national team players	(GPS) units to track kinematics and mechanical efforts. Individual CK values were measured every morning.	Players could be classified based on their sensitivity to micromovements (MM), high velocity (HV), or a combination of the two.	93.3
(86)—Football	24 male professional players (27 ± 9 years, 79 ± 15 kg).	Large-Sided games (LSGs) were monitored. TD/(m)min, HSR/(m)min, Sprinting/(m)min, ACC/DEC(n)/min were assessed by using the GPS. RPE was also registered.	Despite LSGs being replicated and sometimes exceeding some match-specific intensity parameters, HSS and sprinting were consistently lower than official matches.	100
(90)—Football	49 elite male professional players.	ACC/DEC (n), Dynamic load stress (a.u.) HMLD (m), HSR (m), SPD (m), DT (m), and PL (a.u.) were assessed by using the GPS.	Players presented different PL across the microcycle, between training and matches and between positions on the field.	96.7
(87)—Football	25 male professional players (27 ± 9 years, 78 ± 14 kg)	Sided games were categorized (LSGs; Medium SGs, and SSGs). EL (HSR, sprinting distance, ACC, DEC) and IL (RPE) were compared by positions. GPS units.	Position differences for HSR, sprinting, and DEC. Sided games promote different RPE, DT, Sprinting (m), ACC, and DEC. MSG promotes higher ACC and DEC.	100

### 3.7. Applicability of the tracking system related to injuries

With regard to the analysis related to injury prediction, some kinematic variables such as distance covered and mechanical ones such as accelerations/decelerations were considered. For example, in elite female FH players, the authors verified that the risk for in-game knee injury decreased with the increasing distance covered while running at low speed (30). In the same path, Caparrós et al. (29), in a total of 2,613 observations and 246 games from 33 different players of a professional male BK team, described that athletes with less or equal than three decelerations per game and those running less or equal than 1.3 miles per game had a higher risk of injury. Predictive injury models are also proposed by the use of tracking systems (7). In this study with 26 Italian professional male players, the authors propose a multidimensional approach to injury forecasting in professional FTB based on GPS measurements (7). In addition, three studies from contact team sports (AUF and AMF) presented associations between specific levels of kinematic and mechanical variables with the occurrence of injuries (11, 99). Finally, some studies reported that lower workloads during preseason and higher workloads during competition training weeks increased the risk of non-contact injuries (100; Nobari et al., n.d.) (Table 3).

**Table 3:** Articles predominantly related to injuries.

Study and sport in which the study was carried out	Sample	Main outcomes measured	Results	Quality score (%)
(30)—Field Hockey	32 Elite players from the Korean Female National Team: 11 defenders, nine midfielders, and 12 forwards. Goalkeepers were excluded.	GPS monitoring was used to record the distance traveled while running at various speeds, sprinting time, and top speed in 20 international competitions. Non-contact injuries with knee and ankle pain were documented.	Low-intensity running distance was considerably greater among athletes who did not suffer an injury during the course of the trial. The risk of in-game knee injury decreased as the distance covered while running at low speed increased.	86.7
(29)—Basketball	2,613 observations and 246 games from 33 different players of a professional male basketball team	Injuries that occurred during regular-season games were reported. Tracking data (speed and distance traveled, mechanical load variables, and locomotor factors) were recorded.	Athletes who experienced less than three DEC per game and ran less than 1.3 miles per game were at a higher risk of injury.	86.7
(7)—Football	26 elite male players (age = 26 ± 4 years; height = 179 ± 5 cm; body mass = 78 ± 8 kg).	Training workload by the GPS: Two Features-Total Distance (dTOT) and HSR Distance (dHSR), and three features-Metabolic: Distance (dMET), HML Distance (dHML), and HMLD Distance per minute (dHML/m).	Distance traveled (DT) can detect approximately 80% of the injuries with nearly 50% precision. The injury forecaster results in a cumulative f1-score = 0.60 on the injury class; In an evolutive scenario, the features chosen modify as the season progresses.	73.3
(99)—Australian Football	45 elite players from one club (age, 22 ± 3 years, height, 190 ± 7 cm; mass, 89 ± 8 kg)	Running efforts using GPS units: absolute, predefined speed criteria or relative, individualized speed thresholds. Players were separated into three equal groups: (1) faster, (2) moderate, and (3) slower. Non-contact injuries were documented.	Slower players with increased relative extremely high-speed running had a higher risk of injury, and greater absolute speed ACWR indicated an increase in injury, whereas greater relative high-speed ACWR promoted a decrease in injury.	93.3
(101)—Australian Football	26 elite male professional players (mean ± SD: age 22.8 ± 3.3 years, range 18–30 years, height 187.1 ± 7.2 cm; body mass 85.8 ± 7.4 kg)	EL by GPS units [distance (m), SPD (>7 m-s-1(m), ACC [3–15 m-s-2(n)], DECC (−3 to −15 m-s-2) (n), PL (a.u), and impacts >3 g (n)]. Creatine Kinase [CK] levels were tested before and after each match.	CK increased in competition. Impacts and game time were most strongly associated with postmatch CK. DEC, ACC, impacts, and SPD are strong predictors of CK. CK is an indicator of muscle damage during competition, with impacts and HIR traits being the best predictors.	100
(11)—American Football	115 players participated in the 2014–2015 season, and 117 participated in the 2015–2016 season.	GPS units were used to determine PL. All injuries sustained during practice or games were documented.	Injuries are associated with greater increases in workload. Individuals with an ACWR ratio greater than 1.6 are 1.5 times more likely than time- and position-matched controls to experience an injury.	86.7
(100)—Gaelic Football	25 male elite-level players (*n *= 25; age 25 ± 3.7 years, height 182.2 ± 6.2 cm; body mass (BF) 86.7 ± 7.8kg; %BF 12.7 ± 3.6%)	TD, PL, and meters covered at different running speeds were collected through GPS data. All injuries sustained during practice or games were documented.	Players who completed <50% of preseason sessions had a greater probability of non-contact injuries. Increased preseason running loads may lower the risk of injury while also improving or preserving aerobic fitness.	100
(12)—Field Hockey	14 professional female players (age: 20.4 ± 5.4 years; body mass: 60.7 ± 7.2 kg; height: 167.0 ± 1.0 cm)	Muscle strength, GPS data (TD; ACC/DEC intensity level, and player wellness during and after matches using various tools such as s-RPE, GPS units, 5-WQ, and TQR.	Players experienced decreased hip strength and acute fatigue after the second postmatch, with prolonged reduced strength in the non-dominant limb. Fatigue levels returned to normal after 48 h.	86.7
(84)—Football	21 professional players aged 28.3 ± 3.9 years.	Acute workload (AW); Chronic Workload (CW); ACWR; and Increment in acute workload (*Δ*-AW) were estimated through EL data by using GPS units throughout the training period and during matches.	Workload and the occurrence of non-contact injuries have a good association, according to high load weeks.	93.3
(102)—Football	33 male professional players 25.9 ± 3.8 years; 182.1 ± 6.9 cm; 74.2 ± 6.7 kg	GPS units to assess kinematic (TD, HSR) and mechanical (ACC/DEC) metrics. Injuries were also assessed.	Over a 4-week period, a considerable increase in players’ weekly external load performance may have an adverse effect on the occurrence of injuries.	93.3

### 3.8. Applicability of the tracking system related to nutrition

Two studies that analyzed the variation in physical enhancement through nutritional intake were identified. Before and after the players received 140 mL of beetroot juice (BJ) or placebo (PLA) on two separate days, a sample of 10 competitive male BK players was monitored (27). To determine physical improvements, a specific neuromuscular battery of tests followed by a 40 min-simulated BK match was used to measure the players’ running profile, ACC, and DEC; however, acute moderate doses of BJ (12.8 mmol of NO3-) were not effective in improving neuromuscular performance (27). In the other study, the same author verified that the ingestion of moderate doses of caffeine enhanced several physical variables such as jump height, running profile, number of body impacts, ACC, and DEC in FTS professional players (28) (Table 4).

**Table 4:** Articles predominantly related to nutrition.

Study and sport in which the study was carried out	Sample	Main outcomes measured	Results	Quality score (%)
(27) Basketball	10 male basketball players (15.6 ± 0.5 years, body weight 76.3 ± 9.0 kg, height 184.3 ± 7.5 cm, body mass index 22.5 ± 2.9).	Participants drank either beetroot juice or a placebo and then played a simulated basketball game for 40 min. Physical metrics were tested afterward: jump, grip strength, agility, and sprinting. Kinematics/mechanical metrics were monitored.	Acute moderate doses of BJ (12.8 mmol of NO3−) were useless in improving the players’ performance.	93.3
(28)—Futsal	16 top-level male players (age: 28.0 ± 4.1 years; height:173.8 ± 6.1 cm; body mass: 71.2 ± 9.7 kg; futsal experience: 12.4 ± 2.9 years)	Futsal-specific tests (CMJ, 20-m sprint test, kicking velocity, and accuracy test). Simulated matches were conducted between the controlled (ingested 3mg/kg of caffeine) and placebo groups. Kinematics/mechanical metrics were monitored.	Jump, sprint performance, high-speed running, ACC, and DEC, were improved by 3 mg/kg of coffee. Caffeine supplementation improves futsal performance when used in moderation.	100

## 4. Discussion

**Figure 5:**
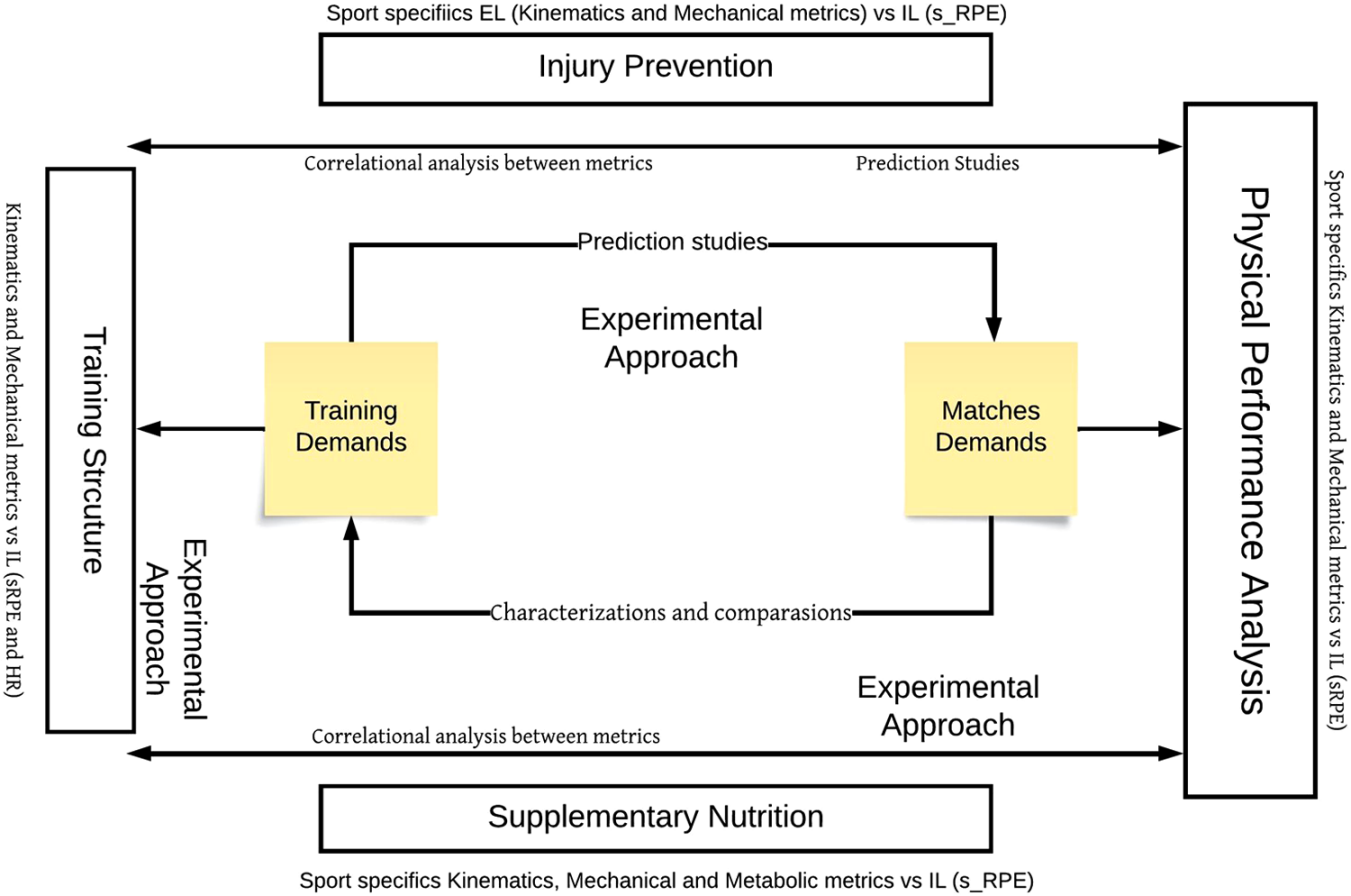
Flow chart of the contextual research applicability of tracking systems based on the screened domains.

The purpose of this scoping review was to understand the applicability of tracking systems in physical performance analysis in team sports over the last decade in order to gain an understanding of how manipulating different variables and research goals in this field may be beneficial for enriching the use of this technology and, as a result, for integrating the revised information in a comprehensive framework that could help clarify the physical performance area for team sports and athlete wellbeing. The main findings were that tracking technology has been applied in team sports as a valuable tool in the following research areas: (1) performance analysis of match demands; (2) performance analysis of training vs. match information; (3) injuries, and (4) nutrition. In addition, the groups of metrics mainly used are (1) kinematic and mechanical; (2) kinematic; (3) kinematic, mechanical, and metabolic. To seek an understanding of athletes’ performance, several studies establish correlational analysis between specific EL metrics and IL information. However, the described EL metrics are manipulated individually rather than in an integrative model, which should include, for example, the principal component analysis (PCA) of each team sport. This indicates that it is still promising to go further into the understanding of how tracking technology can be used to improve training and performance by combining IL and EL data. Finally, studies appear to focus primarily on (1) characterization and comparison of drills, training, and match sessions, followed by (2) correlational analysis between metrics. In line with this, it was possible to consider such relationships for the development of a conceptual framework that supports both applied sport-sciences research and practitioners’ monitoring and intervention (Figure 5). Indeed, the creation of high-performance departments that combine *slow-thinking* (applied sport-science research) and *fast-moving* intervention (applied monitoring and intervention) (103) supported by a coherent framework certainly promotes best preparation assessments and decision-making based on reliable data (103).

### 4.1. Tracking technology and physical performance analysis of match demands and its comparison with training demands

In team sports, coaches and performance personnel invest a lot of time and resources to follow up on their athletes. Physical training can be described in terms of EL (work) and acute responses and long-term adaptations (IL) (104). Through the development of wearable technology for athletes over the past few years, team sports stakeholders have embraced the practice of monitoring their athletes' EL in both training and competition (54). Consequently, technology is used by coaches and exercise physiologists with the intent of enhancing training data and achieving competitive advantage (54). The focus on athlete performance improvement has been driving the need to understand training and match demands. Several researchers have been characterizing and understanding EL metrics (mainly kinematic and mechanical) during specific training or competitive contexts (32, 59). In addition, and as an attempt to enhance knowledge for training improvement, links have been established between kinematic, mechanical, and metabolic variables, and more recently with IL (86, 87). However, and assuming the physiological adaptations of different team sports exigencies, we suggest that stronger steps must be taken based on experimental approaches seeking to better understand the relationship between EL and IL and athletes’ recovery cycles (50, 53). In this regard, we suggest that studying Large-, Medium-, and Small-sided games (LSGs, MSGs, and SSGs) may be helpful in determining which format is most suited to load specific kinematic or mechanical loads in line with a desired IL (86, 87). Positioning, intragames, level of competition, and tactical comparisons have also been pointed out as references for the understanding of how performance may be improved by better understanding EL metrics, particularly in terms of frequency, duration, and intensity. In addition to the difficulties of collecting data at a top-elite level, in order to create a training program that accurately reflects the physiological demands of the game, we suggest that more elite team sports game-specific data are required. In indoor high-intermittent team sports and based on the metrics selected by Ohmuro et al. (75), in order to understand more accurately the demands of games, we suggest a monitoring of EL by effective playing time (in-game). Despite the fact that an agreement between coaches and athletes on overall RPE and sRPE, as well as RPE and sRPE, in moderate and hard categories, has already been recorded (13), there is a lack of information about how demanding is the playing time, by players’ interchange rotation, particularly in indoor intermittent team sports, and how can that information benefit the training structure, and consequently, team performance.

Team sports performance is regarded as highly complex and multivariate. Researchers integrate players' technique with EL and IL, while there are studies in which EL and IL are categorized by players’ position, exploring the differences between training and competition (73, 75, 98). This correlational approach seems to translate differences that are assessed for a better individual and collective preparation, as reported by Florian et al. (105),. When a selected EL fits with sports specification, we suggest that this type of method is reliable for better interpretation of competitive microcycles. As proposed by Ispyrlidis et al. (98), in FTB, and verified by Fernández et al. (47), in RH, this information may combine the interchanges of matches with competitive profile training and improve all parameters of players’ physical output. At the same level, research indicates that an understanding of load dynamics during training and competition, such as players' intra- and interweek fluctuations, exercises a substantial influence on improving elite athletes’ performance by properly regulating workload and recovery (82). In this review, it is found that several researchers in their studies have developed a longitudinal understanding of load dynamic (combined kinematic, mechanical, and metabolic metrics) across training and competition, proposing that performance enhancement may be a reflection of a proper adjustment of specific metrics (46, 67).

More recently, performance analysis research is stepping forward on the understanding of HIAs and on predictive studies for fatigue and match performance. The literature suggests that during different phases of a training cycle, such as the baseline or competition phase, training loads must be modulated to either enhance or reduce fatigue levels (89). In the light of this, it is important to understand the match HIA in order to manage fatigue properly, matching both variables to adaptation to training and for achieving competitive performance (10, 40). On the other hand, understanding HIAs and their impacts on IL also improves the knowledge of individual players’ recovery strategies (89). The importance of understanding effort categories (moderate and hard) has already been established in a systematic review, in which agreements between coaches and athletes about total RPE and s_RPE were reported (13). Therefore, in order to effectively adjust the volume, frequency, and intensity of the metrics that are more closely associated with game pick periods, this information can assist in developing novel approaches on tapering training strategies. In this sense, kinematic and mechanical variables are statistically manipulated with indirect IL such as RPE, suggesting that it is possible to predict fatigue from GPS/LPS training and match data. In addition, a dissociation between EL and IL units may reveal the state of fatigue of an athlete; therefore, detecting changes with statistical approaches may provide the necessary confidence for implementing changes (10). Nevertheless, in contact team sports such as AUF, AMF, RG, and GF, the use of mechanical metrics seems to be more significant to understanding players’ fatigue and muscle damage (32, 39, 43, 71).

Strength and conditioning coaches may daily obtain reports with more than 100 variables (106). Thus, it is crucial to choose and identify the key performance indicators according to sports specifications (63). In this review, we identified research focused on proposing models for match performance mainly based on kinematic, mechanical, and metabolic variables (44, 60–62, 99). These studies demonstrate how crucial it is to adopt statistical techniques like PCA in team sports to accurately use significant performance variables by excluding the least significant ones.

### 4.2. Tracking technology and injuries

Athletes compete fiercely to become the greatest in their respective sports, particularly at the highest level. In team sports, the individual or collective success of this process is often compromised by the occurrence of injuries (30). Therefore, in this review, we observed that the development of injury prevention programs is being supported by the association of tracking positioning systems such as the use of a GPS. Recently, studies tried to understand which (EL metrics) and how (volume, intensity, and frequency) training and game demands have a higher association with the occurrence of specific injuries such as the risk for in-game knee injury (30). In specific intermittent team sports such as FH and BK, low kinematics (low-intensity running) (30) and mechanical (DEC) are associated with a higher risk of injury (29). However these studies do not explain whether this lower EL results from acute or chronic adaptations, such as the one by Li et al. (11), which applied the use of technology to understand the relationship between soft tissue injury and training workload in AMF. For this author, the variation of training stimulus may induce a higher risk of injuries. Once studies have shown that the ACWR is a more accurate predictor of injury than the total workload (18, 101) it is highly advised to use tracking technology to determine an athlete's chronic workload. According to the screened manuscript (7, 11), we suggest that the assessment of an athlete's physical activity based on the intensity, duration, and number of actions of kinematic, mechanical, and metabolic metrics may also predict injuries with acceptable precision. Recently, a higher preseason workload, such as a running performance, was related to a lower risk of injuries (100). On the other hand, higher levels of weekly chronic workload (ACWR) indicate greater chances of sustaining non-contact injuries (84, 91). We suggest that these dichotomic reports may support the need for better longitudinal use of tracking systems in different team sports, in order to establish causal correlations between specific parameters, and consequently, to improve injury prevention measures. The sparse research on the use of tracking technology related to injury forecast is mainly noticed in outdoor team sports; thus, it is recommended that future studies extend to high-intermittent indoor team sports such as FTS, handball (HB), RH, and BK. Finally, there is no injury forecast method that links the most demanding scenarios (MDS) or HIAs in team sports according to specific EL variables and IL information. We suggest that characterizing the dynamic of training workload related to teams' sports MDS may contribute to more reliable specific injury predictions.

### 4.3. Tracking technology and nutrition

The use of nutritional supplements and ergogenic aids to improve performance is becoming more popular among intermittent sports (107), but few have demonstrated benefits (108) such as creatine, caffeine, sodium bicarbonate, and beta-alanine. Indoor team sports (BK, FTS, HB, RH, or netball) are becoming more intense, with explosive demands (37, 47) stronger athletes, and repeated combinations of short and long intermittent efforts (97). This review noticed almost no research on the use of tracking technology to measure nutritional impact in kinematic, mechanical, or metabolic metrics. In this review, only two studies were screened. With regard to the experimental approach developed to understand whether the nutritional supplement of beetroot juice had some impact on the monitored EL variables (27), no significant results were found. Furthermore, the effects of supplementation on physical performance and match running load or true exposure during a competition (total distance covered, speed achieved, number of accelerations and decelerations) in team sports are either sparse or unknown (27). In their recent work, López-Samanes et al. (28) demonstrated how the application of this technology could advance the experimental approach to athletes' nutritional development. The authors showed that 3 mg/kg of caffeine enhanced the physical variables associated with FTS, such as jump and sprint performance, and also improved kinematics (high-speed running) and mechanics (ACC/DEC) during a simulated FTS match. This finding can be interpreted as an opportunity for researchers not only to provide experimental proof of performance enhancement, but also to better understand the role of supplementary nutrition in an athlete's recovery process. To the best of our knowledge, there are no experimental studies aimed to understand athletes' recovery cycles with the influence of supplementary nutrition and monitored by tracking technology.

### 4.4. Limitations

The fact that this review only included papers published in English in the electronic databases of PubMed, Web of Knowledge, and Scopus may have led to the exclusion of relevant publications in other languages. Furthermore, the exclusion of all systematic reviews can be seen as a constraint because some categories related to the aims of this scoping review may not have been identified because of this exclusion.

### 4.5. Actual trends and future directions

Currently, research on the use of tracking systems is mainly focused on the physical performance analysis on matches, training vs. matches, followed by injuries, and nutrition. Both kinematic and mechanical variables combined, and then only kinematics, appear to be the primary observed metrics. In addition, we verified that in some studies, IL information is combined solely with kinematic or mechanical metrics. Few studies combined both metrics. These manipulated strategies used for integrating IL information with EL may have been established according to team sports specifications. Still, we suggest that tracking and studying kinematic, mechanical, and IL parameters together in training and matches would provide more accurate data for the purpose of enhancing our understanding of athletes’ performance and, consequently, to better manipulate the structure of drills, for example, the manipulation of LSGs, MLGs, and SSGs, according to specific physiological responses.

Based on the main results screened in this review, further research and practice should consider how frequency, volume, and intensity may be adjusted according to the optimal (1) training structure; (2) to promote injury prevention; (3) to consider nutritional supplements; and (4) to promote physical performance analysis. The integration of these four dimensions has not yet been proposed. Consequently, how can performance analysis lead to the process of enhancing athletes' performance? This integrative vision proposal illustrates the significance of the domains explored in this review and how they may be oriented in accordance with specified research methodologies for both outdoor and indoor team sports (Figure 5). Despite the existing studies, we consider that the combined information of training and match demands remains the main source of the physical performance analyses. However, the integrative and longitudinal contribution of the presented domains related to the proposed researched strategies may help produce more reliable and accurate information, leading to an enhancement of the training structure. The development of more correlational analyses between metrics or prediction studies will improve the understanding of athletes’ performance enhancement. Also, experimental and prediction studies, particularly in the domain of injury prevention and nutrition, as well as in the manipulation of the metrics measured and compared between training and match sessions, are highly recommended. Therefore, because of its impact on high levels of muscle damage, as well as neuromuscular and perceptual fatigue, longitudinal tracking of EL variables, including kinematic, mechanical, and metabolic metrics, when combined with IL, can provide insights into injury prediction and prevention measures. In addition, the application of tracking technology to nutrition in team sports is relatively unexplored, with limited research investigating the impact of nutritional supplements on such metrics. This emphasizes the current trends in research, the need for more integrative models combining EL and IL data, and the importance of experimental and prediction studies to advance the understanding of team sports' physical performance, injury prevention, and nutritional impact. Ultimately, the integration of these domains into a coherent framework can guide both applied sports science research and practical interventions, leading to an improvement in athlete preparation and decision-making based on reliable data, which may promote the implementation of new drills, training, or competitive designs.
